# Drones providing live video to dispatch centers during time-critical incidents

**DOI:** 10.1186/s12873-026-01752-z

**Published:** 2026-08-28

**Authors:** M. Kristiansson, M. Andersson Hagiwara, L. Svensson, S. Schierbeck, Y. Khalid, P. Lundgren, C. Magnusson, P. Nordberg, J. Hollenberg, M. Ringh, S. Forsberg, A. Claesson

**Affiliations:** 1 Centre for Resuscitation Science, Department of Clinical Science and Education, Karolinska Institutet, Södersjukhuset, Stockholm Sweden; 2 Innovation Platform, Sahlgrenska University Hospital, Region Västra Götaland, Gothenburg, Sweden; 3 Prehospen—Centre for Prehospital Research, University of Borås, Borås, Sweden; 4 Division of Prehospital Care, Region Västra Götaland, Regionhälsan, Gothenburg, Sweden; 5 Emergency Medical Services, Sahlgrenska University Hospital, Region Västra Götaland, Gothenburg, Sweden; 6 Center for Digital Health, Sahlgrenska University Hospital, Region Västra Götaland, Gothenburg, Sweden; 7 Department of Thoracic Surgery and Cardiology, Sahlgrenska University Hospital, Region Västra Götaland, Gothenburg, Sweden; 8 Institute of Medicine, Sahlgrenska Academy, University of Gothenburg, Gothenburg, Sweden; 9 Department of Physiology and Pharmacology, Section on Intensive Care, Karolinska Institute, Stockholm, Sweden

**Keywords:** Drone, EMDC, Video, Assessment

## Abstract

**Background:**

Early triage of emergency calls at dispatch centres relies mainly on verbal information, which may not reflect the complexity and severity of a situation, limiting dispatchers situational awareness and early decision-making. Real-time live-video could potentially be provided by drones before the arrival of emergency medical services and fire department. The aim was to evaluate feasibility and operational value of automated beyond visual line of sight (BVLOS) drones delivering early live-video to emergency dispatch centres during time-critical incidents.

**Methods:**

This prospective interventional feasibility study was conducted in the region of western Sweden between 7th May–7th November 2025. One drone system with automated external defibrillator and a high-resolution camera was integrated into regional medical/fire dispatch centers, covering a 141 km^2^ suburban area (population approx 63 000) enabling real-time videofeed during emergency 112-calls. The drone was dispatched to consecutive alerts meeting predefined criteria (out-of-hospital cardiac arrest, traffic accidents, fires). Outcomes included time delays, characteristics of drone- and video transmission together with dispatcher-perceived value assessed through surveys.

**Results:**

Of 169 alerts, the drone system was operational in 120 (71%). Within this operational population, the drone was deployed in 56/120 alerts (47%). Eleven alerts were subsequently cancelled following updated dispatch information, leaving 45 completed alerts in which the drone arrived on scene and delivered live video to the dispatch centres. The drone arrived first or was the only resource on scene in 44/45 completed alerts (98%), with a median dispatch-to-arrival time of 4:24 min compared with 8:50 min for responding ground units (*p* < 0.001). Video was transferred to dispatch centres in all (100%) completed alerts and was uninterrupted in 91%. Emergency medical dispatchers reported low/moderate value in 79% of incidents, and high value in 21% (*n* = 33), with a median score of 3.0 (IQR 1.0–5.0) on a 0–10 scale. Fire dispatchers reported low/moderate value in 33% and high value in 66% (*n* = 6), median 7.0 (IQR 5.5–9.3) on the same scale. No adverse events were reported.

**Conclusion:**

Deployment of a BVLOS drone to diverse time-critical emergencies is operationally feasible and demonstrates a clear arrival time advantage over ground responders. Perceived operational utility varies between dispatcher groups, with fire and rescue services deriving the greatest tactical benefit from early aerial situational awareness. These findings indicate potential for future drone-integration into emergency response systems.

**Supplementary Information:**

The online version contains supplementary material available at https://doi.org/10.1186/s12873-026-01752-z.

## Background

Emergency medical service (EMS) systems worldwide are facing increasing strain due to demographic changes, rising emergency call volumes, and limited growth in available resources [1–3]. Ambulance response times have increased over time in many settings, reflecting growing demand and system-level challenges, and delays in time‑critical conditions such as out‑of‑hospital cardiac arrest (OHCA), trauma, fires, and drowning are associated with increased morbidity and mortality [4–7]. These incident types are also characterised by high uncertainty at dispatch, limited initial information, and the need for coordinated multi-agency response, making early situational awareness particularly important.

Emergency dispatch centres play a central role in early prioritisation and resource allocation.

In Sweden, the national emergency number (112) handled more than 3.7 million emergency calls in 2025 across police, fire, and emergency medical services, resulting in approximately one million ambulance dispatches [8]. Early decision-making at dispatch relies primarily on verbal information from callers, which may not adequately reflect the complexity and severity of the situation [9, 10]. This uncertainty often leads to overtriage, where additional resources are dispatched to ensure patient safety, potentially contributing to inefficient resource utilisation in high-demand systems [11]. At the same time, complementary response models such as community first responders and the dispatch of fire and police services have been implemented to reduce time to first intervention [12–14]. 

Visual information provided during emergency 112- calls has been proposed as a method to improve dispatcher situational awareness and decision-making [15, 16]. Most existing approaches rely on bystander smartphones or fixed cameras and have shown potential to improve assessment and reclassification of incidents [16–20]. However, these methods depend on bystander availability, may distract from lifesaving actions, and are limited to situations where a caller or camera is present at the scene. While previous studies have demonstrated the feasibility of transmitting still images from drone systems to dispatch centres [21], real-time live video may provide greater situational awareness through continuous assessment of scene dynamics and context [22]. 

Drones are regularly used by fire and police services after arrival on scene to assess hazards and scene extent, and studies in search and rescue scenarios demonstrate improved situational overview [23–26]. Drones have also been used effectively at the site of large public events to identify incidents and guide first responders [27, 28], and drone cameras may as well be used to locate individuals submerged under water in drowning cases [29, 30]. However, these applications are typically conducted within visual line of sight (VLOS) and are not integrated into the early dispatch phase.

Recent studies in Sweden have shown that automated drones flying beyond visual line of sight (BVLOS) can deliver automated external defibrillators (AEDs) and even arrive at the scene of suspected OHCA before EMS in most cases. [31–33]. In addition, drone systems have demonstrated the ability to provide dispatch centres with still images prior to the arrival of ground units [21]. 

These findings suggest a potential role for drone-based visual information earlier in the alarm chain, prior to the arrival of ground units.

However, it remains unclear whether automatically dispatched BVLOS drones can provide real-time live video to emergency dispatch centres during ongoing incidents, and how such early visual information may support situational awareness, decision-making, and resource allocation at the dispatch level. This represents an important gap in the early phase of emergency response.

The aim of this study was to evaluate feasibility and operational value of automated beyond visual line of sight drones delivering early live-video to emergency dispatch centres during time-critical incidents.

## Methods

### Study design

This was a prospective interventional feasibility study evaluating one (1) automated BVLOS drone system providing live video to two different emergency dispatch centres i.e. regional (a) medical dispatch centre and (b) fire department dispatch centre, during time-critical incidents.

The study assessed operational feasibility, time performance, technical performance, and dispatcher-perceived usefulness at two occasions (a) immediately after alert and (b) at follow up, one month post study period.

### Setting

**Fig. 1:**
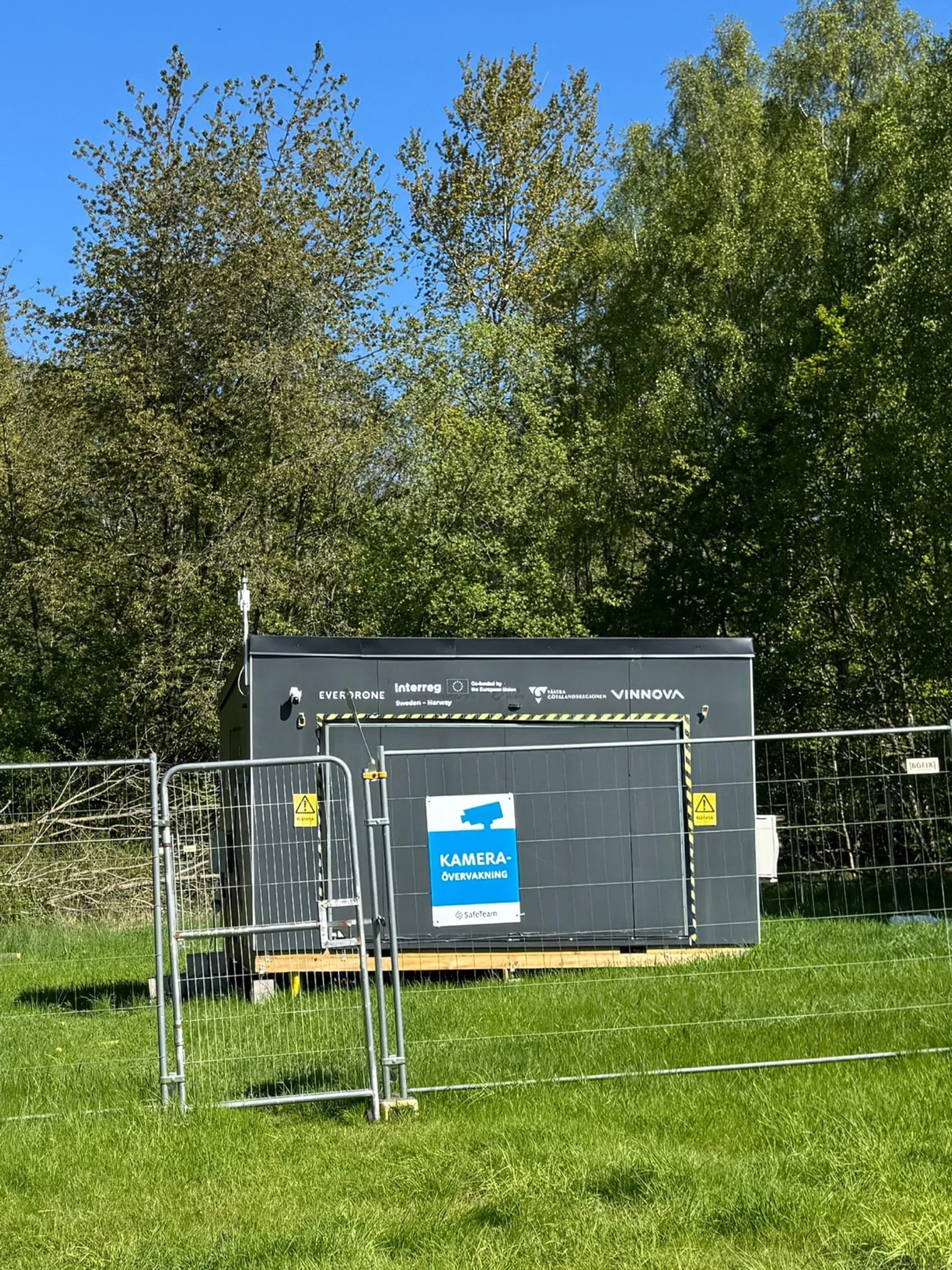
Automated drone system with hangar, Gothenburg, Sweden 2025. Automated drone hangar (2 × 5 m) with surrounding fencing. Equipped with power, heating and Wi‑Fi, enabling autonomous launch and recovery. The hangar features automatically deploying doors and supports QR‑code–assisted automated landing after completed missions. Photo: Andreas Claesson

The study was conducted in the Gothenburg area in th region of west Sweden during a 6-month study period between the 7th of May to the 7th of November 2025. One AED and camera-equipped drone system was placed in a suburban area south of Gothenburg integrated in a fully automated hangar (Fig. 1), for remotely operated BVLOS flights.

The drone covered a predefined geographic area of 141 km² with an estimated population of 63 000. The area was annotated in terms of topography, cell towers, no flight zones and separation to high rise buildings. The drone system was fully integrated with the EMDC, the Helicopter Emergency Medical Service-coordinator (HEMS), the regional fire and rescue departments dispatch centre and with local EMS and aviation authorities. The system was operational daily between 08:00 and 22:00. Operating hours were determined by the operational permissions and practical staffing conditions governing the drone system during the study period. Incidents occurring outside these hours were not eligible for drone deployment. All aviation safety procedures were followed with regards to permits and regulations from the Swedish transportation agency for BVLOS operations according to SAIL 2 standards [34]. 

### Drone system and cameras

The drone platform used in this study was an upgraded version of an already operational automated AED‑delivery system (Everdrone AB, Sweden), configured for BVLOS operations. The underlying flight automation, navigation, and safety architecture of the system have been described in detail in previous studies evaluating drone‑delivered AEDs in real‑world out‑of‑hospital cardiac arrest scenarios. [21, 32, 33]

**Fig. 2:**
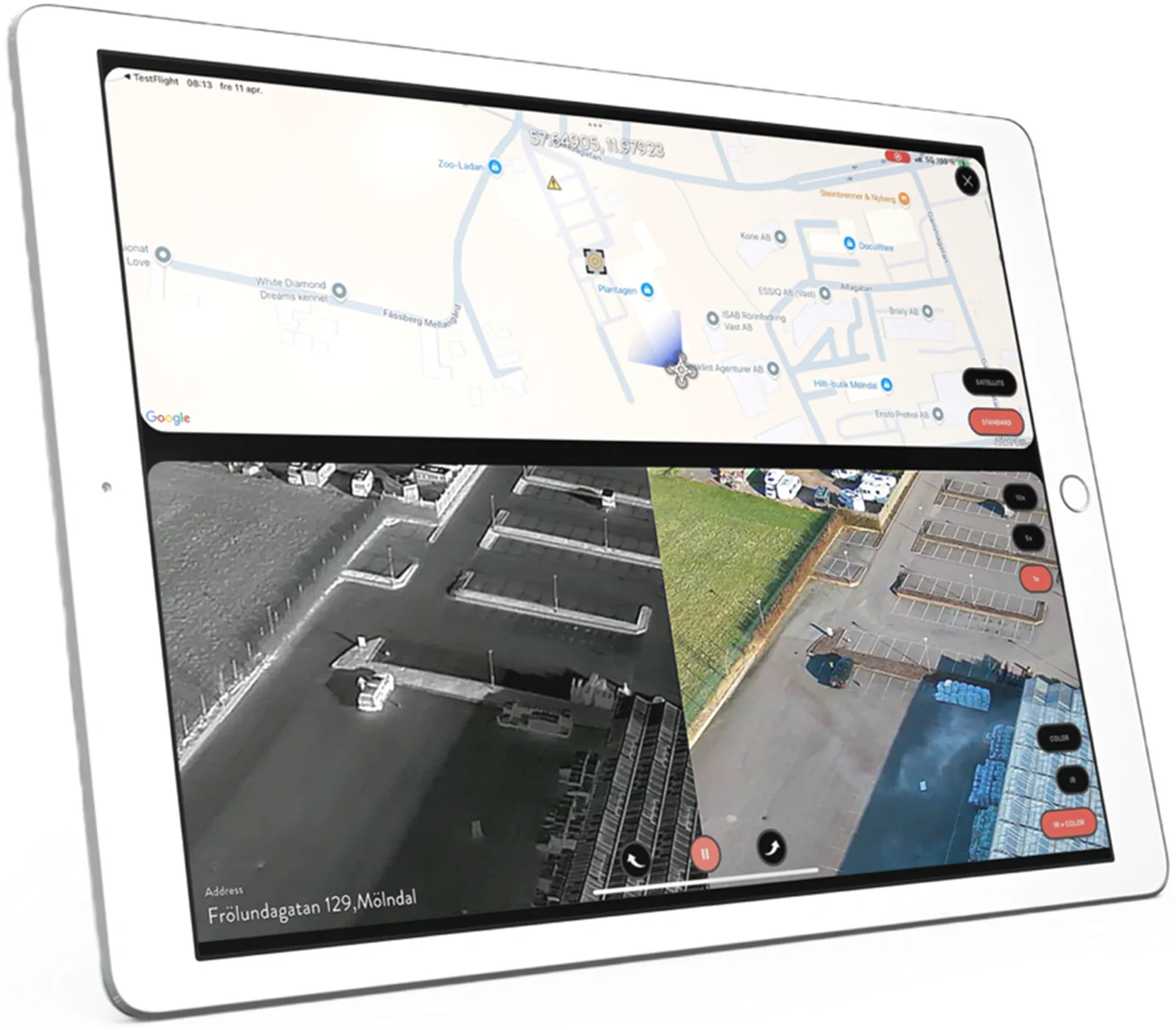
iPad with mission control app for providing situational awareness at the dispatch centre. iPad with mission control application for situational awareness at the dispatch centre. The application streams live drone video to the EMDC and displays a three‑pane interface: map view, thermal imaging (bottom left), and RGB camera feed (bottom right), enabling early situational awareness during ongoing incidents. Dispatchers can zoom and remotely steer the camera via the iPad. Photo: Everdrone AB

For the present study, the system was enhanced to support real‑time transmission of live video to emergency dispatch centres. The drone carried two functionally distinct camera systems. A forward‑facing navigation camera was used exclusively by the drone operator to ensure safe flight, obstacle avoidance, and precise navigation. This camera was not accessible to dispatchers and served no clinical or operational assessment purpose. (Fig. 2)

**Fig. 3:**
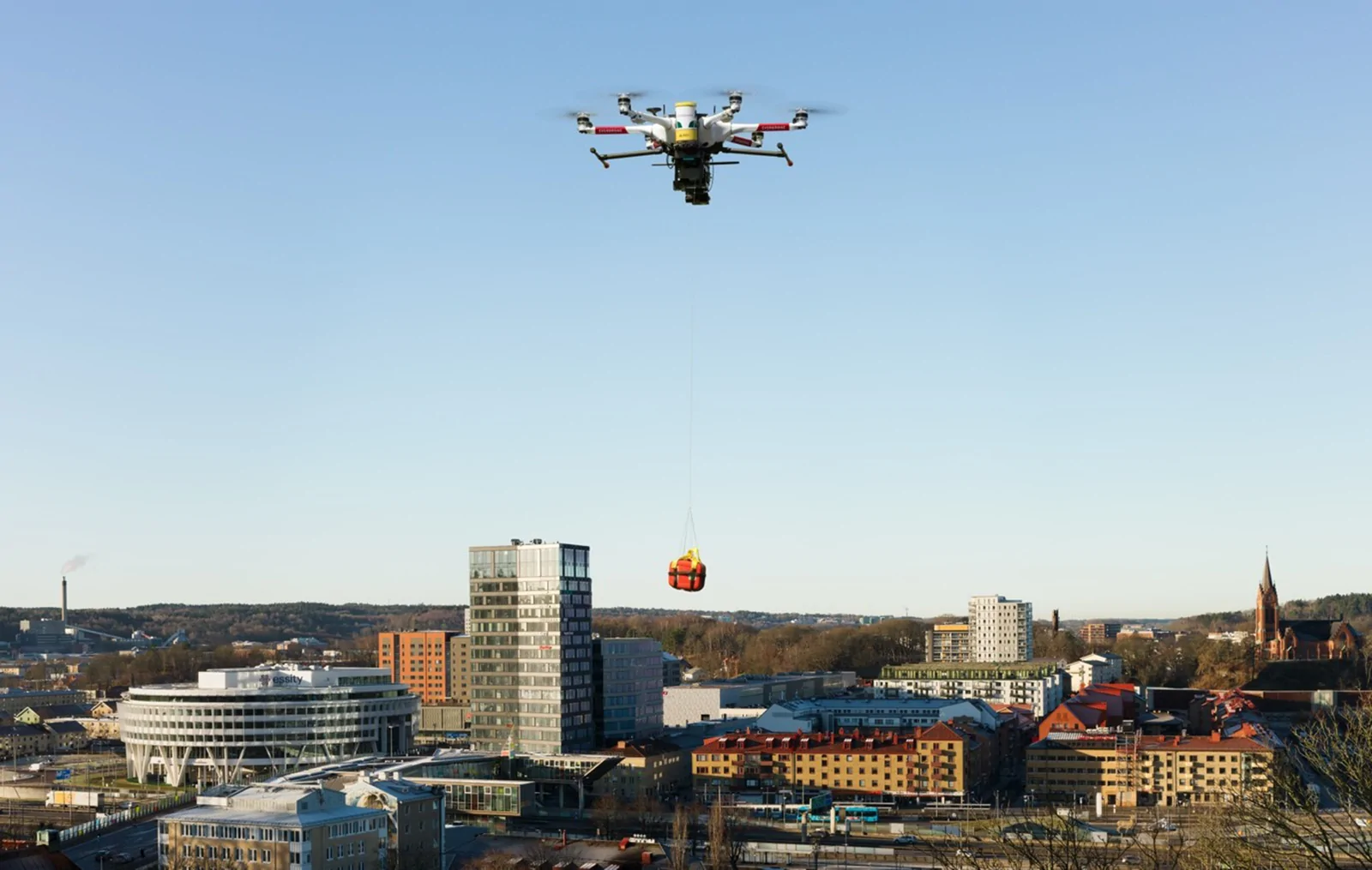
Custom drone equipped with AED and high-resolution camera. The Everdrone E2 UAV is based on the airframe Dark Matter hX Gen2b, developed by Clogworks Technologies Limited. Maximum take-off weight for the E2 UAV is 17 kg. The drone has a cruise speed of approximately 80 km/h and an operational range of up to 5 km. Photo: Everdrone AB

Mounted beneath the drone was a dedicated payload sensor module, consisting of a high‑resolution optical RGB camera with zoom capability and thermal imaging. This payload camera system was designed specifically for situational awareness at the incident site and was remotely controlled by the EMDC. At the emergency medical dispatch centre, dispatchers were able to adjust camera orientation and zoom in real time via a dedicated application on a stationary iPad positioned at the HEMS coordinator workstation. A second iPad at the fire dispatch centre provided video reception only. Dispatchers could review the video feed while simultaneously managing the emergency call without interacting with the flight control system. (Fig. 3) The remote pilot and the dispatcher were not co-located and operated from separate locations. Flight control remained under the responsibility of the remote pilot and mission control system, while dispatchers could only control the camera payload through the application. In addition to camera orientation and zoom, dispatchers could indicate points of interest on the screen, request drone rotation around the scene, or instruct the system to maintain a stationary hover position.

Live video transmission was initiated automatically at take‑off and streamed continuously to the EMDC and the fire and rescue dispatch centre throughout the mission. Video and command‑and‑control communication were transmitted over the cellular network using four independent modems with separate SIM cards, enabling simultaneous connectivity via multiple mobile network operators. This configuration provided redundancy, improved bandwidth availability, and reduced the risk of connection loss during flight.

The communication link was encrypted, and all safety‑critical flight functions were handled onboard the drone, ensuring continued safe operation even in the event of degraded network connectivity. No action from callers or bystanders at the scene was required to establish or maintain the video feed.

The drone system was dispatched for two primary purposes:

- delivery of an AED in suspected out‑of‑hospital cardiac arrest, and.
- provision of early live video during time‑critical incidents, including traffic accidents, fires, drowning events, and other dispatcher‑identified emergencies.

### Incident selection and dispatch process

The drone system was integrated with the regional EMDC, enabling automated alerts for predefined time‑critical incident categories occurring within the drone’s operational area. When an emergency call to the national emergency number (112) met predefined index and geographic criteria, a drone alert was generated automatically. Dispatchers could also manually request drone deployment when early situational awareness was considered potentially valuable.

Upon alert, the system performed an automated pre‑flight assessment, including airspace availability, weather conditions, system status, and regulatory constraints. If no exclusion criteria were met, the drone was launched.

After take-off, the drone navigated autonomously along a dynamically calculated flight path. A certified remote pilot supervised the flight in accordance with aviation regulations, while emergency dispatchers monitored the live video feed and controlled the payload camera to assess the scene during the ongoing emergency call.

Live video transmission was initiated automatically at take‑off and remained available until mission termination or return‑to‑base. Missions were classified as completed when the drone arrived on scene and the live video feed was successfully activated. Missions could be cancelled after take-off if updated information from the emergency call indicated that drone support was no longer required.

### Inclusion criteria for drone take off

**Fig. 4:**
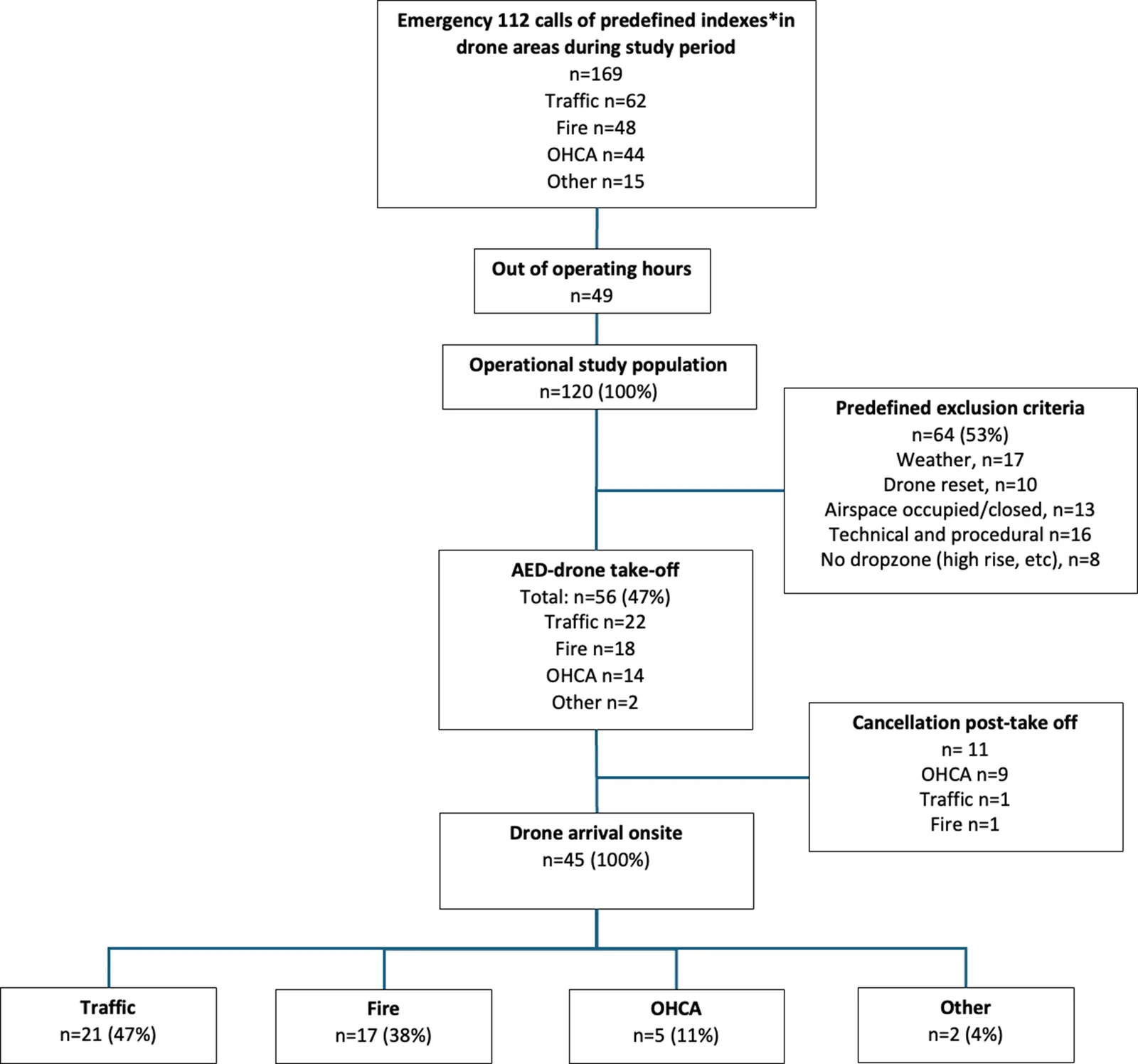
Flow chart - relay of live drone video to the dispatch centre during time dependent-incidents over 6 months, May 2025- November 2025. Flowchart of drone dispatch to emergency calls during the study period, including eligible alerts, exclusions, drone deployment, cancellations, and completed missions by incident type

- Emergency calls to the national emergency number (112) occurring within the predefined drone operational area.
- Incidents classified under predefined time‑critical dispatch indexes, including:
  - suspected out‑of‑hospital cardiac arrest (OHCA),
  - traffic accidents (including railway),
  - fires (building, vehicle, or terrain),
  - drowning or person in water,
  - other dispatcher‑identified incidents where early situational awareness was considered potentially valuable.
- Incidents occurring during system operating hours; 8:00–22:00.
- Successful completion of automated pre‑flight checks without safety or regulatory conflicts. (Fig. 4)

### Exclusion criteria for abortion of mission

- Alerts received outside system operating hours.
- Weather conditions exceeding predefined safety limits (e.g. wind, precipitation, icing risk).
- Airspace restrictions or conflicts preventing safe BVLOS operation.
- Technical constraints including time to restore the drone after completed mission.

Missions cancelled after take‑off due to updated information from the emergency call indicating that drone support was no longer required.

### Data sources

1. EMDC emergency 112 call logs (PSAP and regional dispatch with HEMS-c): characteristics in terms of indexes, timestamps, call categories, resource allocation.
2. Drone system logs (Everdrone AB): characteristics in terms of route allocation, weather conditions, flight data, technical status, arrival times, video metadata.
3. Video recordings from completed missions: Scene characteristics (e.g. number of individuals, visible hazards, scene extent and environmental factors) were obtained through post hoc review of recorded drone video by the research team.

### Survey procedures

For documenting perceptions and experiences from the dispatchers of receiving live video from incident sites using drones, the survey was distributed on two separate occasions:

1. Incident-level survey -immediately after alert.

- Participants: All EMDC HEMS coordinators and fire department dispatchers who received and viewed the live video feed.
- Administration: The survey was made available for completion as soon as possible after each incident where video was received through a digital link / QR-code.
- Content: Video quality, added information, decision impact, communication, workflow effects (Appendix A).

2. Follow-up survey – one month after study closure.

- Participants: All participating dispatchers who provided survey-answers at incident level.
- Administration: The survey was administered through a digital link/QR code and completed one month after study closure.
- Content: The follow‑up survey focused on overall perceived usefulness, informational value beyond the emergency call, perceived impact on decision‑making, and general reflections on workflow and operational value. (Appendix B).

### Questionnaire development and pre-testing

The questionnaire was developed by the research group specifically for this feasibility study. Items were selected to align with the predefined study objectives and to capture dispatchers’ self-reported experience of: (i) video-streaming performance and technical disruptions; (ii) whether the live video confirmed or added information beyond that obtained during the emergency call; (iii) the perceived influence of the video on actions, decision-making, and resource deployment; and (iv) perceived operational value. The draft questionnaire was informally pre-tested with a small number of intended users before study initiation to assess comprehensibility and perceived relevance. This informal pre-testing did not constitute a formal pilot study or structured cognitive interviewing procedure. No formal evaluation of content validity, construct validity, reliability, or other psychometric properties was performed. Responses comprised categorical and ordered categorical response options, a single 0–10 numerical rating item, and free-text questions. The complete questionnaire is provided in Appendix B.

### Outcomes

**Drone system performance** was assessed with focus on operational feasibility. This included the proportion (%) of all incoming alerts that resulted in actual drone take-off, the proportion (%) of alerts completed successfully with the drone arriving on scene, the proportion (%) of alerts with successful live video transmission to EMDC, alongside time delays for drone, EMS and fire department respectively. Time from dispatch-to-arrival and drone time benefit on scene was compared to the first responding ground unit EMS and fire department.

**Dispatcher experiences** encompassed the perceived usefulness of the live video during individual incidents, whether the video contributed with additional information beyond what was conveyed in the 112 calls, and the degree to which the video influenced operational decision-making. Additionally, assessments were also made whether the drone’s video feed enabled dispatchers to locate the incident site based on aerial imagery. The surveys further explored how the video affected workflow, communication between EMDC and fire services, and the overall perceived value of integrating early drone-based situational awareness.

### Statistics

Categorical variables are presented as counts and proportions (%) at group level. Time intervals are presented as medians with interquartile ranges (IQR). Descriptive statistics were calculated using Microsoft Excel (version 16.107.4). Differences in self-reported survey responses between groups, as well as differences in response times, were analysed using the Mann–Whitney U test. All tests were two-sided, and p-values < 0.05 were considered statistically significant.

### Ethical approval

Ethical approval was sought from the Swedish Ethical Review Authority (2022-06624-01; 2024-06559-02). All participating dispatchers gave written informed consent. Video feeds showed non-identifiable overviews only and all data were stored securely.

In addition, permission for camera surveillance in connection with healthcare operations was obtained from the Swedish Authority for Privacy Protection. This authorisation (case number IMY-2024-10072) granted legal approval for the use of aerial video recording during time-critical medical incidents within the framework of the study, in accordance with Swedish camera surveillance legislation and data protection requirements.

This research has been conducted in accordance with the declaration of Helsinki.

## Results

### Overview of alerts and missions

During the six-month study period, 169 emergency alerts met predefined criteria for potential drone deployment. Of these, 49 alerts (29%) occurred outside system operating hours and were therefore not eligible for drone deployment, leaving 120 alerts constituting the operational study population.

Among these 120 alerts, 64 did not result in drone deployment due to weather conditions (*n* = 17), airspace restrictions (*n* = 13), technical or procedural factors (*n* = 16), drone reset requirements (*n* = 10), or lack of a suitable drop zone (*n* = 8). Consequently, the drone system was successfully deployed in 56/120 alerts (47%) (Fig. 4).

Following deployment, 11 alerts were cancelled after take-off because updated information indicated that drone support was no longer required. The remaining 45 alerts were completed, with the drone arriving on scene and delivering live video to the dispatch centres.

Most completed alerts were related to live‑video use in outdoor incidents such as traffic accidents and fires *n* = 40/45 (89%), while 5/45 alerts (11%) involved suspected OHCA.

No adverse events related to drone operations were reported.

### Drone operational performance

Across all completed alerts, the drone demonstrated rapid and consistent response times. Median dispatch‑to‑arrival time was 4:24 min (IQR 3:36–5:05) as compared to first arriving ground unit, EMS or fire department with 8:50 min (7:46–11:22) p = < 0.001. The drone arrived first or was the only resource on scene in 44 of 45 alerts (98%). (Table 1)

**Table 1:** Drone operational performance and response times

Variable	Drone (*n* = 45)	First ground responder (*n* = 45/27)
Time from 112 call to dispatch, median min: sec (IQR)	0:52 (0:37–1:15)	0:57 (0:34–1:29)
Dispatch-to-arrival time, median min: sec (IQR)	4:24 (3:36–5:05)	8:50 (7:46–11:22)
Arrived first on scene, n (%)	44 (98%)	1 (2%)
Lead time (drone vs. ground), median min: sec (IQR)	2:51 (1:47 − 6:45)	-
Drone only resource on scene, n (%)	18 (40%)	-
Distance to incident, median km (IQR),	3.0 (2.2–3.8)	-

Ground response times were calculated only for cases where a ground unit arrival time was available (*n* = 27). In the remaining cases, the drone was the only resource on scene

In 18/45 alerts (40%), no ground unit arrival was recorded, and the drone was the only resource observed on scene during the mission. These cases primarily involved traffic accidents (12/18, 67%) and fire-related alerts (5/18, 28%), with one additional OHCA case (1/18, 6%). These incidents were often later assessed as low severity or non-events upon aerial review. (Table 2))

**Table 2:** Drone operational performance by incident type

Variable	Traffic (*n* = 21)	Fire (*n* = 17)	OHCA (*n* = 5)	Other (*n* = 2)
Drone dispatch-to-arrival, median min: sec (IQR)	4:21 (3:55–4:51)	4:02 (3:25–4:55)	4:59 (4:24–5:01)	5:39 (5:28–5:50)
Ground dispatch-to-arrival, median min: sec (IQR)	7:04 (6:01–9:51)	7:48 (6:59–10:16)	7:33 (5:54–9:33)	13:25 (10:36–16:13)
Lead time, median min: sec (IQR)	2:45 (1:45–6:53)	3:05 (1:51–6:35)	4:22 (2:18 − 6:04)	7:16 (4:29–10:03)
Drone arrived first, n (%)	8/9 (89%)	12/12 (100%)	4/4 (100%)	2/2 (100%)
Drone only resource on scene, n (%)	12 (57%)	5 (29%)	1 (20%)	0 (0%)

Arrival‑time comparisons showed that when time stamps for both drone and ground units were available, the drone arrived before the first ground unit in 26 of 27 cases (96%). (Table 1)

### Incident categories

- **Suspected OHCA**: A total of 44 suspected OHCA alerts occurred during the study period. The drone system was dispatched in 14 of these alerts (32%). In 9/14 (64%) of the dispatched alerts, the mission was cancelled after take‑off based on updated information during the emergency call, resulting in 5/14 (36%) completed OHCA alerts where the drone arrived on scene and delivered an AED, in these cases with a median time benefit of 4:22 min (IQR 2:18 − 6:04). All OHCA incidents occurred indoors and outside the camera field of view, precluding assessment of patient condition or resuscitation efforts. No incident‑level survey responses were obtained for OHCA missions.
- **Traffic accidents**: Totally 62 traffic‑related alerts were identified, of which the drone completed 21/62 (34%) alerts. In more than half 12/21 (57%) of these alerts, the drone was the only resource on scene. When ground arrival data were available, the drone arrived first in 8/9 (89%) of cases, with a median time benefit of 2:45 min (IQR 1:45 − 6:53).
- **Fire‑related alerts**: Among 48 fire‑related alerts, the drone completed 17 alerts. In all cases with comparable arrival times, the drone arrived before ground units, with a median lead time of 3:05 min (IQR 1:51 − 6:35). Nearly one‑third 5/17 (29%) of fire missions involved the drone as the sole resource on scene.
- **Other incidents**: 15 alerts were classified as other incidents, including drowning‑related, explosion and unclear outdoor events. The drone completed 2/15 (13%) of these alerts. In both cases where ground arrival data were available, the drone arrived first (2/2, 100%), with a median lead time of 7:16 min (IQR 4:29–10:03). No other‑incident missions involved the drone as the sole resource on scene, and both cases were later assessed as non‑events upon aerial video review. Table 2.

### Observed scene characteristics from live video

Observed scene characteristics were derived from post hoc review of recorded drone video. Across all 45 completed alerts, the video provided an aerial overview of the incident scene upon arrival. Observable characteristics included the number of individuals present outdoors, general movement patterns (e.g. standing or lying persons), vehicle positions in traffic accidents, and visible smoke or fire development in fire-related incidents.

In traffic‑related alerts, the video commonly enabled assessment of vehicle location, traffic flow, and presence of persons inside or outside vehicles. In fire‑related alerts, the video allowed identification of smoke plumes, visible flames, and the approximate extent of the affected area. No patient‑level clinical assessments were possible in suspected OHCA missions, as all such incidents occurred indoors and outside of the camera field of view. See appendix videos 1 and 2.

### Dispatchers’ experiences – incident‑level surveys

Incident‑level survey responses from EMDC dispatchers were available for 36 of the 45 completed alerts (80%). Out of these, video quality could be assessed in 33 incidents, and the live video feed functioned without technical disturbances in 30/33 (91%).

EMDC dispatchers reported low/moderate value in 26/33 (79%) of incidents and high value in 7/33 (21%) with a median value score of 3.0 (IQR 1,0–5,0) on a 0–10 scale. The video feed most often confirmed information already obtained during the emergency call (20/33, 61%), while new information was identified in a smaller proportion of cases (7/33, 21%).

Fire and rescue dispatchers submitted incident‑level responses in 6 incidents, all corresponding to alerts already assessed by EMDC dispatchers. In contrast to EMDC responses, fire dispatchers reported higher perceived value, with a median score of 7.0 out of 10 (IQR 5,5–9,3) on a 0–10 scale, with low/moderate value in 2/6 (33%) and high value in 4/6 (66%). Among fire dispatchers, the video feed both confirmed information from the emergency call (4/6, 67%) and provided new information in 3/6 (50%) of cases. Table 3.

**Table 3:** Dispatchers Incident-Level Survey Responses after event

Variable	EMDC responses (*n* = 36)	Fire department responses (*n* = 6)
Dispatchers’ availability to view video	33/36 (92%)	6/6 (100%)
Video stream transferred to dispatch centres *, n (%)	33/33 (100%)	6/6 (100%)
Video stream transferred without disruptions*, n (%)	30/33 (91%)	6/6 (100%)
Confirmed information from emergency 112-call*	20/33 (61%)	4/6 (67%)
Number of cases providing new information *, n (%)	7/33 (21%)	3/6 (50%)
Perceived added value score 0–10, of live-video, median (IQR)*	3.0 (1.0–5.0)	7.0 (5.5–9.3)
Perceived added value of drone-delivered situational awareness, n (%) *		
No value (0) Low/moderate (1–5) High (6–10)	0/33 (0%) 26/33 (79%) 7/33 (21%)	0/6 (0%) 2/6 (33%) 4/6 (66%)
Communication between dispatch centres worked well	11/33 (33%)	5/6 (83%)

* *n* = 33. 3 missing who did not have time to view video

### Follow‑up survey

In the follow‑up survey conducted one month after study closure, EMDC dispatchers (*n* = 7) reported moderate perceived value (median 5.0), primarily related to confirmation of information from calls. Fire and rescue dispatchers (*n* = 12) reported substantially higher value (median 8.0), frequently citing new information regarding incident extent and hazards. See Table 4.

**Table 4:** Dispatchers Survey Responses at Follow up (post-study period)

Variable	EMDC dispatchers (*n* = 7)	Fire dispatchers (*n* = 12)
Gender		
Female Male	4 (57%) 3 (43%)	4 (33%) 8 (67%)
Years of experience		
0–2 years 2–5 years 6–10 years More than 10 years	1 (14%) - 1 (14%) 5 (72%)	1 (8%) 1 (8%) 3 (25%) 7 (63%)
Perceived added value score 0–10, of live-video, median (IQR)	5.0 (4.0–7.0)	8.0 (7.0–9.25)
Confirmed information from emergency 112-call	4/7 (57%)	10/12 (83%)
Identification of new information n (%)	2/7 (29%)	8/12 (67%)
Perceived added value of drone-delivered situational awareness, n (%)		
No value (0) Low/moderate (1–5) High (6–10)	0/7 (0%) 4/7 (57%) 3/7 (43%)	0/12 (0%) 2/12 (17%) 10/12 (83%)
Impact on decision-making (any)		
None Low (a little) Moderate High	2 (29%) 5 (71%) 2 (29%)	7 (58%) 2 (17%) 2 (17%) 1 (8%)

Follow-up survey responses from emergency medical (EMDC) and fire dispatchers one month after the study period, including demographics, experience, perceived value of live video, and reported impact on decision-making

## Discussion

A key finding of this study is the consistent time advantage of drone deployment, with the drone arriving before or as the only resource on scene in nearly all completed alerts. This highlights the potential of automated BVLOS drones to provide earlier situational awareness than traditional ground-based response. Across 45 completed alerts, the drone arrived first or was the only resource on scene in nearly all cases, with dispatch‑to‑arrival times comparable to or faster than those reported in previous first‑responder drone studies [21, 32, 33]. The system therefore appears technically mature and operationally robust in a real‑world, mixed‑incident environment. However, the relatively low proportion of completed alerts underscores the need for optimisation to increase applicability. Potential improvements include extended operating hours, reduced airspace constraints, enhanced dispatch integration, and more targeted activation criteria. The time required to restore the drone to operational readiness between consecutive missions was not recorded in this study and should be evaluated in future large-scale implementations where simultaneous or closely spaced incidents may occur.

The present concept differs from both smartphone-based video solutions and responder-operated drone systems. Unlike smartphone-based video, drone-delivered video does not require active participation by callers or bystanders during an ongoing emergency and therefore avoids diverting attention from potentially life-saving actions. Furthermore, visual information can be obtained even when no caller remains at the scene or is able to operate a mobile device. Unlike systems where drones are launched after responders arrive on scene, the drone in this study was dispatched as an independent resource directly from the emergency dispatch centre. This enabled visual situational awareness during the earliest phase of the incident and frequently before arrival of ground units.

The relatively low proportion of completed alerts should be interpreted in the context of previous early-stage drone implementations. It is also important to distinguish between deployment feasibility and completed missions. The operational study population consisted of 120 alerts occurring during periods when the drone system was available for deployment. Within this population, the drone was deployed in 56 cases (47%). Eleven alerts were subsequently cancelled after take-off because updated information indicated that drone support was no longer required. These cancellations reflected changes in incident assessment rather than technical failure or inability of the drone system to operate. In a recent study evaluating dispatch of drones for transmission of still images to emergency dispatch centres, drones were deployed in only 13% of eligible alerts, primarily due to airspace restrictions and weather limitations [21]. In comparison, the higher deployment proportion observed in the present study suggests that advances in system integration, automation, and operational procedures may substantially increase applicability over time.

Several factors limiting drone deployment were potentially modifiable. Operating hours were restricted to 08:00–22:00 as part of the operational framework governing the system during the study period. Although this reduced overall system coverage, it reflected the practical conditions for evaluating a new dispatch-integrated drone service. Future implementations may benefit from extended availability and evaluation under 24-hour operating conditions. In addition, temporary airspace restrictions and technical or procedural constraints, including maintenance activities and system reset requirements, contributed to reduced deployment rates. Future developments in automation, system integration, and airspace management may increase operational availability and improve deployment rates.

Integration with other airspace users remains an important consideration for future implementation. Although no conflicts with helicopter emergency medical services occurred during the study period, larger-scale deployment will require robust procedures for coordination with manned aircraft and other airspace stakeholders. Continued development of airspace management solutions, including U-space concepts, may facilitate such integration while maintaining operational safety.

Another finding is that the apparent operational value of early aerial video was not uniform across organisations. EMDC dispatchers perceived low but meaningful benefits, with added informational value in roughly one‑third of incidents and limited influence on decision‑making. In contrast, the fire and rescue dispatch centre consistently reported substantially higher usefulness, both in incident‑level surveys and in the overall follow‑up survey. Fire dispatchers frequently identified new information related to incident extent, scene layout, and potential hazards, and they reported moderate or high influence on tactical decisions in several missions. These divergent perspectives likely reflect structural differences in operational roles: EMDC dispatchers focus primarily on caller interrogation and medical triage [35], whereas fire dispatchers are responsible for tactical resource allocation and therefore benefit more directly from rapid visual assessment when managing early resource allocation and tactical decisions [36]. These findings should be interpreted cautiously given the limited number of incident-level responses.

In suspected out‑of‑hospital cardiac arrest, no incident‑level survey responses were obtained, precluding assessment of dispatcher‑perceived usefulness of the live video. All OHCA missions occurred indoors and outside the drone’s field of view, which limited the ability to visually assess patient condition or ongoing resuscitation efforts. OHCA was nevertheless retained as an important incident category, as it provides a stringent test of response time performance in one of the most time-critical emergency conditions. Consequently, the role of early drone‑based video in supporting dispatch decision‑making during OHCA could not be evaluated in this study and should be interpreted primarily as a technical feasibility finding rather than an assessment of clinical or operational value. However, video to support handling of OHCAs has been examined in several studies, primarily using smartphones [15–19], but also retrospectively with the use of CCTVs [20]. These studies show a significant value in using visual aid in OHCA treatment.

Despite the technical performance of the system, inter‑agency communication emerged as a notable barrier. Two‑thirds of EMDC incident‑level responses reported difficulties communicating with the fire and rescue dispatch centre, whereas none of the fire‑dispatch respondents reported communication problems. This asymmetry may stem from differences in staffing models, task loads, and communication pathways rather than flaws in the drone system itself. Still, the absence of a shared video interface was repeatedly mentioned and likely contributed to the perceived communication burden at EMDC. In the present study, live video was available only to the dispatch centres. Future systems may allow selected visual information, or the video stream itself, to be shared directly with responding units while en route. This could potentially enhance situational awareness before arrival and further support operational decision-making.

Beyond individual incidents, the findings suggest a potential system‑level benefit of early aerial video in improving dispatch specificity. In a substantial proportion of completed missions, the drone was either the first or the only resource on scene, and several non‑OHCA incidents were assessed as low‑severity or non‑events upon aerial review. Such early confirmation of limited incident severity may support decisions to avoid unnecessary escalation or secondary resource mobilisation, thereby reducing system strain in environments characterised by high call volumes and limited response capacity. While this study was not designed to measure downstream effects on resource allocation, the results indicate that real‑time visual information early in the alarm chain may contribute to more efficient prioritisation and resource management within emergency response systems.

Several studies have discussed the potential psychological effects on emergency dispatchers of receiving visual information from incident scenes, as visual access may reduce professional distance and bring dispatchers closer to traumatic events compared with traditional audio‑only emergency calls [16, 17]. While enhanced visual situational awareness may support assessment and coordination, it may also increase emotional strain or cognitive load for dispatchers [18]. The present study did not assess psychological impact or well‑being among dispatchers. However, as live video becomes increasingly integrated into emergency dispatch workflows, further research is needed to explore potential psychological effects and to identify appropriate training and support mechanism [37]. 

It should also be noted that no major or large‑scale incidents occurred during the study period. Such events, characterised by greater uncertainty, wider geographic extent, or competing resource demands, may represent scenarios where early aerial situational awareness provides the greatest operational value [23, 28, 38]. Consequently, the perceived and system‑level benefits observed in this study may represent a conservative estimate of the potential utility of drone‑based live video in emergency dispatch.

Another insight is the drone’s utility in confirming the absence of an ongoing emergency. In two completed missions within the “Other” category, retrospective review of the video revealed no identifiable incident or injured person. Although these missions yielded no survey responses, they illustrate the potential role of drones in resolving uncertainty during ambiguous or low‑information alerts.

The study was not designed to evaluate patient outcomes, triage accuracy, resource utilisation, or objective changes in decision-making, and no conclusions regarding these outcomes can be drawn.

The economic implications and staffing requirements of implementing drone-based situational awareness systems were outside the scope of this study. Future research should evaluate operational costs, personnel requirements, and cost-effectiveness, as these factors will be important determinants of scalability and long-term implementation.

### Limitations

This study has several limitations. First, the number of completed missions was limited, and the study was not constructed to assess downstream effects on clinical outcomes or system‑level resource utilisation. Second, dispatcher‑perceived usefulness was assessed using study-specific, non-validated questionnaire surveys, which may be subject to response bias and differed in administration between organisations. Third, no incident‑level survey responses were obtained for suspected out‑of‑hospital cardiac arrest, precluding evaluation of perceived value in this patient group. Finally, all OHCA incidents occurred indoors and outside the drone’s field of view, limiting assessment of patient condition and resuscitation efforts. These findings should therefore be interpreted primarily as a feasibility evaluation of early live video delivery to dispatch centres rather than a definitive assessment of clinical or operational impact. Finally, the study period did not include any major or large‑scale incidents, limiting direct inference regarding the utility of early drone‑based video in high‑complexity or mass‑casualty scenarios.

The study was conducted between May and November and did not include the Scandinavian winter season. Although missions were performed under varying weather and light conditions, including darkness during evening operations, the system was not evaluated during periods characterized by prolonged winter darkness, snow, or more severe weather conditions. Previous studies using the same drone platform have demonstrated operational capability across a broader range of environmental conditions, including winter operations [32], but the value of live video and thermal imaging under such conditions remains to be evaluated.

Dispatcher-reported outcomes were assessed using a study-specific questionnaire that underwent informal pre-use review but no formal pilot testing or evaluation of its measurement properties. Consequently, measurement error and differences in item interpretation cannot be excluded, and the survey findings should be interpreted as exploratory.

## Conclusion

Deployment of a BVLOS drone to diverse time-critical emergencies is operationally feasible and demonstrates a clear arrival time advantage over ground responders. Perceived operational utility varies between dispatcher groups, with fire and rescue services deriving the greatest tactical benefit from early aerial situational awareness.

These findings indicate potential for future drone-integration into emergency response systems.

## Supplementary Information

Below is the link to the electronic supplementary material.


Supplementary Material 1



Supplementary Material 2



Supplementary Material 3



Supplementary Material 4


## Data Availability

The datasets generated and/or analysed during the current study are not publicly available due to regulations regarding sensitive information and personal data, but are available from the corresponding author on reasonable request, subject to applicable ethical and legal restrictions.

## Abbreviations

AED: Automated External Defibrillator
BVLOS: Beyond Visual Line of Sight
CCTV: Closed-Circuit Television
EMDC: Emergency Medical Dispatch Centre
EMS: Emergency Medical Services
HEMS: Helicopter Emergency Medical Service
HEMS-C: Helicopter Emergency Medical Sevice - Coordinator
OHCA: Out-of-Hospital Cardiac Arrest
PSAP: Public Safety Answering Point
RGB: Red, Green, Blue
VLOS: Visual Line of Sight
