# Supplementary material for: Drones providing live video to dispatch centers during time-critical incidents
Source: BMC Emerg Med. 2026 Aug 28;26:236. doi: 10.1186/s12873-026-01752-z (PMC13531748; doi:10.1186/s12873-026-01752-z)
Supplement: Supplementary file 1 [file 12873_2026_1752_MOESM4_ESM.docx]

**Appendix A**

**Definitions**

- Alert: An event where the system issued an automated or manual drone dispatch signal.
- Decline: An alert where the drone did not take off due to exclusion criteria (e.g., weather, airspace conflict, operating hours).
- EMDC: Regional emergency medical dispatch centre
- HEMS-c: Coordinator for HEMS at the EMDC
- Take‑off: Successful drone launch.
- Cancellation: Mission aborted after take‑off but before arrival.
- Completed mission: Drone arrived on scene and video feed activated.
- PSAP: National emergency dispatch center – SOS Alarm
- Response time (assignment‑to‑arrival): Time from dispatch decision to arrival on scene.
- Lead time: Time difference between drone arrival and first ground responder arrival.
- Only resource on scene: No EMS, fire, or police unit arrived before mission termination.

**Appendix B**

Survey questions for emergency dispatchers

1. **VGRID or employment number**
2. **Which organization do you belong to?**
   a. Emergency medical dispatcher at Region VGR - SVLC
   b. Fire department
3. **What index did the alarm have?**
   a. Traffic accident
   b. Fire
   c. Drowning
   d. OHCA
   e. X?
4. **What case number?**
5. **Was the video streaming successful without disruptions?**
   a. Yes without disruptions
   b. Disruptions
   c. No video feed available at all
6. **What disruption(s) did you experience during the video streaming (if yes on q6)**
   a. The connection did not work at all
   b. The connection was interrupted.
   c. The connection froze
   d. The image was blurred or out of focus
   e. Other
7. **Was the drone able to confirm the information you received in the 112 call?**
   a. Yes
   b. no
8. **Were you able to spot things of value in the video that were not apparent in the call?**
   a. no
   b. Yes, hazards
   c. Yes, extent of event
   d. yes, position
9. **If yes - please describe what you saw in the video that was not clear from the call**
10. **Did the early situational awareness influence your actions and/or decision-making?**
    a. No, not at all
    b. Yes, a little
    c. Yes, to some extent
    d. Yes, a lot
11. **Did the situational awareness lead you to deployment of more or fewer resources/units, recall of resources/units or similar?**
    a. Yes – dispatch of additional units
    b. Yes – dispatch of fewer, or no units at all
    c. No
12. **Please describe how the early situational awareness influenced your actions and/or decision-making or why it did not.**
13. **Did you feel that early situational awareness from drones was valuable?**
    a. 1: No, it just created disturbance 2. No, disturbed a little 3. Neutral, added no value but didn’t disturb 4. Yes, to some extent. 5. Yes, to a large extent**.**
14. **Indicate how value-adding you felt the early situational awareness was. Indicate on a scale of 0-10 where “0” was NOT value-adding and “10” was maximum value-adding.**

| **0** | **1** | **2** | **3** | **4** | **5** | **6** | **7** | **8** | **9** | **10** |
| --- | --- | --- | --- | --- | --- | --- | --- | --- | --- | --- |

15. **Describe why you found early situational awareness valuable or not?**
